# Sonographic Characterization of the Pericruciate Fat Pad with the Use of Compression Elastography—A Cross-Sectional Study among Healthy and Post-Injured Patients

**DOI:** 10.3390/jcm13092578

**Published:** 2024-04-27

**Authors:** Michał Kanak, Natalia Pawłuś, Marcin Mostowy, Marcin Piwnik, Marcin Domżalski, Jędrzej Lesman

**Affiliations:** 1 Department of Orthopaedics and Traumatology, Veterans’ Memorial Teaching Hospital in Lodz, Medical University of Lodz, 90-419 Lodz, Poland; 2 OrtoTeam Clinic, 90-127 Lodz, Poland

**Keywords:** musculoskeletal ultrasound, adipose tissue, knee injury, middle genicular artery, sonoanatomy, echogenicity, elastography, pericruciate fat pad

## Abstract

**Background**: The pericruciate fat pad (PCFP) in the knee joint is still insufficiently studied despite its potential role in knee pathologies. This is the first reported study which aimed to clarify the characteristics of the PCFP in healthy individuals and contrast them with cases of post-traumatic injuries. **Methods**: Conducted as a retrospective cross-sectional study (*n* = 110 knees each) following STROBE guidelines, it employed grayscale ultrasound with echogenicity measurement, compression elastography with elasticity measurement, and Color Doppler for blood flow assessment. **Results**: PCFP showed a homogenic and hyperechoic echostructure. The echogenicity of the PCFP was higher than that of the posterior cruciate ligament (PCL) (*p* < 0.001, z-score = 8.97) and of the medial head of gastrocnemius (MHG) (*p* = 0.007, z-score = 2.72) in healthy knees, but lower than subcutaneous fat (SCF) (*p* < 0.001, z-score = −6.52). Post-injury/surgery, PCFP echogenicity surpassed other structures (*p* < 0.001; z-score for PCL 12.2; for MHG 11.65 and for SCF 12.36) and notably exceeded the control group (*p* < 0.001, z-score = 8.78). PCFP elasticity was lower than MHG and SCF in both groups, with significantly reduced elasticity in post-traumatic knees (ratio SCF/PCFP 15.52 ± 17.87 in case group vs. 2.26 ± 2.4 in control group; *p* < 0.001; z-score = 9.65). Blood flow was detected in 71% of healthy PCFPs with three main patterns. **Conclusions**: The main findings, indicating increased echogenicity and reduced elasticity of PCFP post-trauma, potentially related to fat pad fibrosis, suggest potential applications of echogenicity and elasticity measurements in detecting and monitoring diverse knee pathologies. The description of vascularity variations supplying the PCFP adds additional value to the study by emphasizing the clinically important role of PCFP as a bridge for the middle genicular artery on its way to the inside of the knee joint.

## 1. Introduction

Within the human knee joint, four fat pads exist, three located in the anterior compartment: the infrapatellar fat pad (Hoffa’s fat pad), the anterior suprapatellar fat pad, and the posterior suprapatellar fat pad. Understanding of their anatomy is well-established [1,2,3]. The functions of the fat pads include shock absorption and filling the dead space within the joint. By adjusting their shape during movement, they aid in distributing synovial fluid [1,2,3]. The literature indicates that fat pads contain broadly defined stem cells, acting as a reservoir for these cells within the joint [4,5]. Moreover, adipose tissue also functions as an endocrine organ due to the release of proinflammatory adipocytokines like TNFα, IL-6, IL-8, leptin, and visfatin into the bloodstream [4,6]. These adipocytokines may impact bone and cartilage tissue formation and degradation [1,2,3,4,5]. Additionally, these fat pads contain broadly defined stem cells, acting as reservoirs within the joint [4,5], and are implicated in pain symptoms associated with various knee pathologies [5,7,8,9].

The fourth fat pad, the pericruciate fat pad (PCFP), situated in the knee’s posterior compartment, lacks comprehensive understanding. Consequently, in July 2021, our systematic literature review on the PCFP was published [10]. Notable findings from this review included the description of the PCFP transfer technique in anterior cruciate ligament (ACL) reconstruction or repair, aiding in graft or ligament resynovialization and revascularization [11]. An association was also found between an increased PCFP signal intensity and ACL tears [3]. Furthermore, the relationship between the middle genicular artery (MGA), a primary supplier of knee intra-articular structures, and the PCFP was highlighted. Although the MGA penetrates the fatty structure en route to the joint’s center, limited knowledge about the PCFP prevented further detailed information from being obtained [12,13,14]. Nevertheless, our cadaveric study confirmed this fatty structure as the PCFP [15].

Recent focus has shifted toward fat pads’ reactions to surgical procedures [16], chondral lesions [17], or osteoarthritis [18,19,20,21]. These structures tend to exhibit increased signal intensity in magnetic resonance imaging (MRI) or echogenicity in ultrasound (US) in such pathologies. While MRI has been utilized in examining the PCFP regarding its correlation with ACL injury [3], osteoarthritis (OA) [22], and chondral lesions [23], publications regarding PCFP in ultrasound examination (US) are notably absent. This gap in the literature concerns both ultrasound studies on PCFP’s characteristics in healthy patients and those after trauma. Despite MRI’s advantages, US in orthopedics is a cost-effective, widely available method with fewer contraindications and complications. 

Tissue elasticity changes have long served as a marker of pathology, forming the basis of examinations like palpation [24,25,26,27]. Sonoelastography, surpassing palpation in its precision, is currently employed in various medical areas, such as breast (Tsukuba University Score [24]), thyroid [28] and liver neoplasms (German/Japanese Elasticity Score or LF Index) [27]. Its newer applications extend to the musculoskeletal system, assessing changes due to overuse, trauma, or surgery and encompassing pathologies such as Achilles tendinopathy [26,29], lateral epicondylitis [30,31] or rotator cuff tears [32]. It may also be useful in assessing muscle quality (e.g., muscle stiffness, fibrosis, or fatty degeneration) after ruptures or during myopathies or sarcopenia [25,33]. This technique offers real-time tissue assessment, proving to be quick and cost-effective, to enable dynamic examination, and to lack major contraindications [33]. Assessing tissue stiffness through elastography can serve as a valuable complement to traditional echogenicity measurements in B-mode imaging [33]. 

This study aimed to describe the sonoanatomy of the PCFP, encompassing its echostructure, echogenicity, elasticity, and vascularization among healthy patients. Additionally, it sought to compare the echogenicity and elasticity of the PCFP among healthy individuals and those with post-traumatic conditions. The hypothesis posited that following trauma or surgery, the PCFP would exhibit increased echogenicity and decreased elasticity.

## 2. Materials and Methods

### 2.1. Study Design and Setting

**Figure 1:**
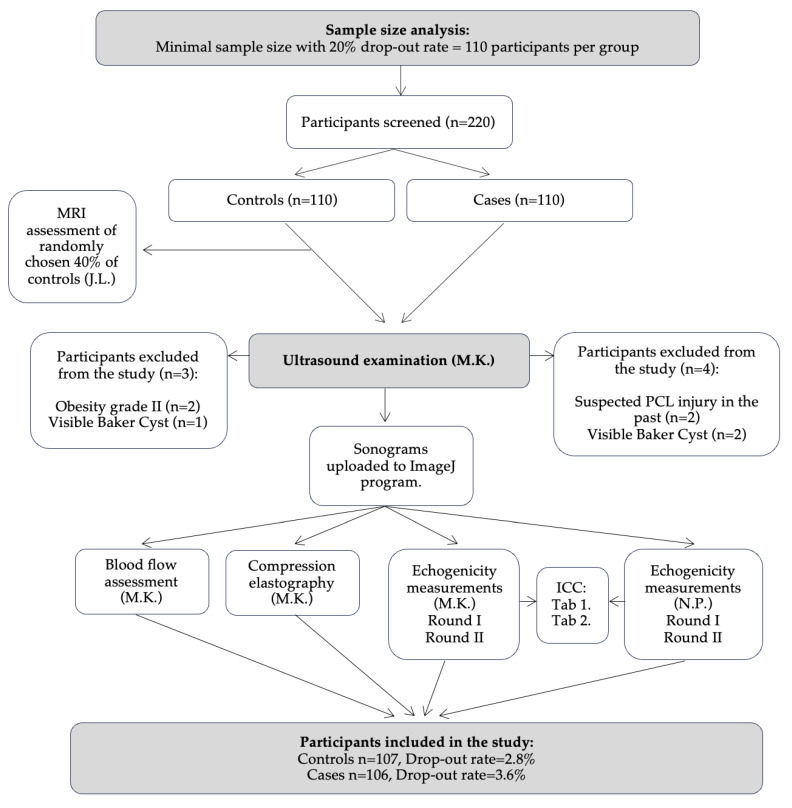
Flowchart presenting the step-by-step methodology of the study. MRI—magnetic resonance imaging, PCL—posterior cruciate ligament, ICC—intraclass correlation coefficient.

This cross-sectional study was set in the orthopedic ward and performed from 8 November 2022 to 3 October 2023. The protocol of the study was prepared and approved by the local bioethics committee (No. RNN/246/22/KE). All steps of the study were conducted in accordance to Declaration of Helsinki [34] and the STrengthening the Reporting of OBservational studies in Epidemiology (STROBE). A checklist for cross-sectional studies was downloaded from www.equator-network.org. A flowchart depicting the main steps of the study is presented in Figure 1.

### 2.2. Participants and Sample Size

The study participants were students of the Medical University of Łódź aged 18–36 years who voluntarily agreed to participate in the study. Informed consent was obtained from all involved subjects. Other inclusion criteria for the control group were the physiological appearance of the knee joint in ultrasound examination; negative results in physical tests; and no history of pain or inflammatory, degenerative, or traumatic conditions affecting the knee joint. Participants with gait disorders, joint malalignment (varus/valgus), or pes planus were excluded. The exclusion criteria for the control group were any gross knee pathology confirmed by anamnesis or physical or ultrasound examination, histories of knee surgery, intra-articular or periarticular injections, or dry needling. Randomly chosen, 40% of the controls had MRI ordered to confirm the healthy status. The inclusion criteria for the case group included meniscal, ligamentous, cartilaginous, or tendinous injuries, at least 3 months post-injury or post-surgery. The exclusion criteria for the case group included conditions that altered the normal sonographic image of the posterior knee or made it challenging to perform ultrasound, such as PCL injury, Baker cyst identified during ultrasound, grade III obesity, and knee flexion contracture.

The following demographic and anthropometric variables were recorded: age, sex, weight, height, and body mass index (BMI). Before undergoing ultrasound examination, each participant underwent a comprehensive physical examination, including physical tests to verify the inclusion and exclusion criteria. The performed tests included: patellar apprehension test, anterior and posterior drawer tests, varus and valgus stress tests, McMurray test, Apley test, and Hoffa’s test. The physical examinations were conducted by two physicians (J.L. and M.K.), one after the other, with blinding between examiners. In case of discrepancies between the results of the physical examinations, the opinion of a third physician (M.D.) was necessary to qualify the participant for the study.

The a priori sample size estimate was based on the results from a pilot study performed on the first 10 knees in each group [35]. This approach was introduced since the effect size could not be determined, due to a lack of previous similar literature. G*Power-3.1 software was utilized (Heinrich-Heine-Universität Düsseldorf, Düsseldorf, Germany) [36]. The outcome measures which were used for power analysis were: (1) echogenicity ratio SCF/PCFP between the groups and (2) elasticity ratio SCF/PCFP between the groups. The effect size for the echogenicity ratio SCF/PCFP between the groups was calculated as 0.71, with the power of 99% and with α = 0.05, and resulted in a minimal sample size of 74 participants in each group. The effect size for elasticity ratio SCF/PCFP between the groups was calculated as 0.64, with the power of 99% and with α = 0.05, and resulted in a minimal sample size of 91 participants in each group. A higher study group value was chosen to keep the power level for both chosen outcome measures. At the end of the analysis, the drop-out rate was predicted as 20% and the minimal sample size was increased to 110 participants per group. The convenience sampling method was used. 

### 2.3. Ultrasound Assessment

The ultrasound device MyLab Seven (Esaote Biomedica, Geneva, Italy) was employed for the examination. A linear transducer SL1543 with a frequency range of 3–13 MHz and a field of view of 14–47 mm was selected. Following the selection of a predefined preset specifically designed for the knee joint, two focus points were positioned to align with the depth of the PCFP. Detailed B-mode parameters were: FR 43; D 44 mm; DR 140; PWR 100; PRC 10/2/2/6; and PRS 0. Detailed elastography parameters were: PRC M/9/7; PRS 3; and P 80%. The examination protocol included a multiplanar ultrasound scan of the popliteal fossa. The patient was placed in prone position with the extended knee exposed for the examination. The positioning of the transducer for visualizing the PCFP was the same as that for PCL visualization described by Bianchi and Martinoli [37]. 

**Figure 2:**
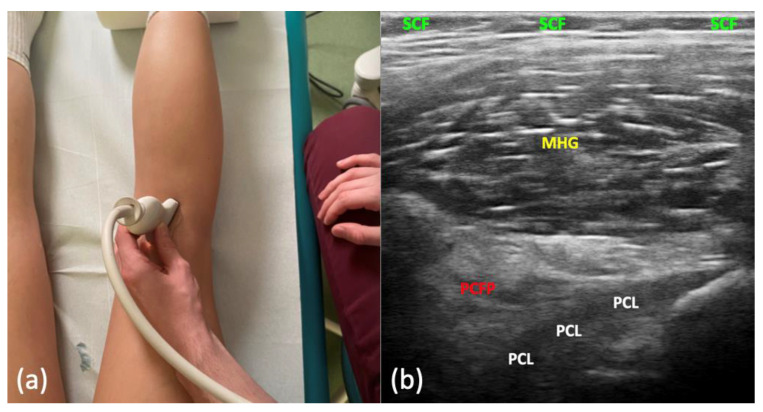
(**a**) Probe positioning. (**b**) Ultrasound longitudinal image of the pericruciate fat pad (PCFP). MHG—medial head of gastrocnemius, PCL—posterior cruciate ligament, SCF—subcutaneous fat.

The protocol commenced with the visualization of the posterior aspect of the medial meniscus in a standard projection. Subsequently, the examiner slightly moved the transducer posterolaterally towards the center of the popliteal fossa, but no further than until the popliteal artery became visible in the image. The near end of the transducer was slightly medially rotated toward the medial condyle of the femur. The transducer was then aligned along the long axis of the PCL (oblique sagittal plane). In this projection, approximately 2/3 of the length of the PCL from the tibial end was visible as a hypoechoic band. Above it, the PCFP was visible as a hyperechoic triangular structure (Figure 2). Two focal points were positioned at the center of the PCFP and at the point where it attached to the joint capsule. The acoustic window of the PCFP was assessed, and differences in its echostructure were compared based on knee position—full extension and at flexion angles of 30°, 60°, 90°, and 120°. Scans were performed in both the longitudinal and transverse planes across the entire surface of the PCFP, from the femur to the tibia. 

The ultrasonographic characterization of the PCFP in the ultrasound examination was established collaboratively by three researchers (M.K., N.P. and M.P.) and subsequently described. The echostructure of the PCFP was evaluated for homogeneity, presence of connective tissue septa, fat tissue lobules, and vascularity. Subsequently, the echogenicity of the PCFP was compared to the surrounding PCL, the medial head of the gastrocnemius muscle (MHG), and subcutaneous fat tissue (SCF). 

### 2.4. Echogenicity

The echogenicity of the PCFP, PCL, MHG, and SCF was determined using ImageJ software (Version 1.53; National Institutes of Health, U.S.; https://imagej.nih.gov/ij/; accessed on 21 July 2021), adapted from the previous literature [21,38,39,40,41]. In the scan depicting the PCFP, PCL, MHG, and SCF in the oblique sagittal plane, the structures were manually outlined using a Free Hand Tool. Subsequently, using a grayscale range from 0 to 255, the number of pixels was measured for each of the 256 possible values for each structure. The obtained values determined the echogenicity of the examined structures. The values were presented as arithmetic means with standard deviations (SD), and then compared inside and between the groups. To establish a more dependable correlation between echogenicity measurements, the SCF/PCFP ratio was introduced in both the control and study groups, as was previously carried out in the literature [32]. Additionally, these ratios were compared with each other. All the echogenicity measurements were considered as continuous variables. Echogenicity measurements were performed twice by two blinded researchers, and the intra- and inter-rater reliability was assessed by calculating the intraclass (kappa) correlation coefficient (ICC). Values lower than 0.5 were defined as indicating poor reliability, those between 0.5 and 0.75 as moderate reliability, between 0.75 and 0.9 as good reliability, and values greater than 0.90 indicated excellent reliability [42]. A multivariable subgroup analysis and post hoc analysis of PCFP echogenicity and SCF/PCFP ratio considering the type of injury (intra-articular, extra-articular, or mixed) was performed in the case group at the end of the analysis.

### 2.5. Blood Flow Assessment

The presence of blood flow in the PCFP was examined using Color Doppler (CD), adapted from the previous literature [38], and it was primarily categorized as a dichotomous variable. During the study, this variable was considered as trichotomous, which is further described in “results” section. The CD parameters that were utilized included: pulse repetition frequency 1.2 MHz; wall filters 4; and Doppler frequency 7.1. The color gain was set at the moment of subsidence of artifacts in the subcortical bone (approximately 50–70%). Flow was assessed in two planes, and if necessary, confirmed using Pulsed Doppler to exclude artifacts. Due to the softness of tissues in the popliteal fossa, special care was taken not to press the transducer head against the skin, which could compress small blood vessels and distort the examination results. The choice of CD over Power Doppler (PD) was based on previous literature. This approach was proposed by Boesen et al. [43], and the preference for CD over PD was also indicated in a similar ultrasonographic study on Hoffa’s fat pad [38]. In case of doubt during the examination (e.g., due to low sensitivity of CD or artifacts), PD was additionally used.

### 2.6. Compression Elastography

**Figure 3:**
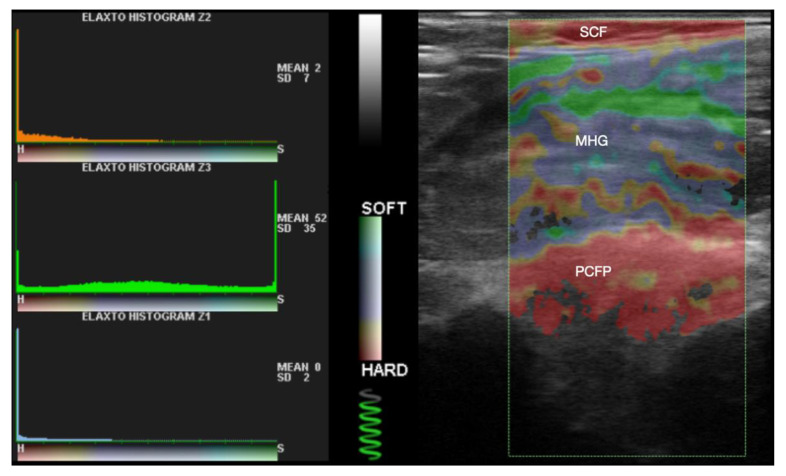
Elastography measurement. Histograms show mean value and standard deviation of a measurement. Z1 histogram pertains to the pericruciate fat pad (PCFP), Z3 to the medial head of gastrocnemius (MHG), and Z2 to subcutaneous fat (SCF). Compression quality icon is visible under the soft/hard color scale.

The evaluation of the elasticity of the PCFP and its comparison with surrounding tissues was conducted using compression sonoelastography and the ElaXto software (version 0.2) integrated into the ultrasound machine. The same software was used in a similar study on Hoffa fat pads by Vera-Pérez et al. [38]. Upon activating the ElaXto function, a reading frame appeared on the screen, the height of which was adjusted to encompass the entire visible depth of the image, and the width corresponded to the dimension of the visible PCFP. The mentioned software calculated the tissue elasticity based on the examiner’s application of slight, repetitive pressure with the transducer on the patient’s skin. Information about the appropriate force and frequency of compressions was displayed on the left side of the screen as the compression quality icon, in the form of a spring filling up with green color. Images with compression quality icons below 3/4 filling were repeated. Quantitative tissue assessment in elastography was made possible by a color scale ranging from red to green with values from 0 to 100. Red represented harder tissue, such as bone or tendon, while green represented softer tissue, such as muscle (Figure 3). All elastographic measurements were considered as continuous variables. First, the assessment of the homogeneity of the PCFP was performed. Following this, a quantitative measurement of the elasticity of manually marked PCFP, MHG, and SCF was performed using the ElaXto Ratio Trace Measurement tool. PCL was not considered in this analysis because, due to anisotropy, the software could not interpret its elasticity. Color scale values for each of the analyzed structures were presented in the form of arithmetic mean with SD. Subsequently, elasticity comparisons were made: PCFP vs. MHG, PCFP vs. SCF in both groups, and PCFP in the control group vs. PCFP in the case group. In agreement with the previous literature [32,33], the elasticity ratio was computed using SCF as a reference, determined as the ratio between the mean SCF elasticity and the mean PCFP elasticity (SCF/PCFP). The arithmetic means of these ratios, along with their standard deviations (SDs), were compared across the groups. A multivariable subgroup analysis and post hoc analysis of PCFP elasticity and SCF/PCFP ratio considering the type of injury (intra-articular, extra-articular, or mixed) was performed within the case group at the end of the analysis.

### 2.7. Magnetic Resonance Imaging (MRI)

To screen for pathologies and ensure compliance with the inclusion/exclusion criteria, 40% of the randomly selected controls underwent MRI examination to confirm the “healthy” status of the knee. A 1.5-T MRI machine (MR5300, Philips Medical Systems, Koninklijke Philips N.V., 2004–2023, Amsterdam, The Netherlands) equipped with a six-channel phased-array knee coil was used. Patients were positioned supine with the knee flexed to 30°. Sagittal and coronal T1W_TSE and sagittal and axial PDW_TSE_SPAIR_Tra sequences were performed (Table 1). One orthopedic surgeon (J.L.), with 8 years of clinical experience, assessed all MRI images. If any uncertainty arose, a second orthopedic surgeon (M.D.), with over 20 years of clinical experience, was on hand for consultation.

### 2.8. Bias

To prevent potential sources of bias, stringent inclusion and exclusion criteria were applied to both cases and controls. Factors such as grade III obesity, posterior cruciate ligament pathologies, age, or osteoarthritis, which could potentially impact the analyzed variables, were carefully considered. There was also a possibility of hypothetical bias being present within the ultrasound examination process and the utilized parameters. The involvement of a single investigator in both the MRI evaluation and the ultrasound examinations would introduce bias by potentially limiting diverse perspectives and cross-validation of findings. However, ensuring that the entire ultrasound examination was conducted consistently by a single researcher (M.K.) using uniform parameters, as previously outlined, provided homogenous sonograms for measurement and prevented other sources of subjective bias. Lastly, the method used for sampling posed volunteer bias; however, it is present in all non-probability sampling methods. 

### 2.9. Statistical Analysis 

Descriptive statistics were assessed for demographic and anthropometric variables, such as age and BMI, using the mean and standard deviation. Gender was treated as a binary variable, and its distribution among groups was analyzed. The number of cases with observed flow in the CD in PCFP was presented as a percentage. Analytical statistics covered the remaining two aspects of the study, namely, echogenicity and elasticity.

Before further analysis, the normality of continuous variables was assessed using the Shapiro–Wilk test, and homogeneity of variance was checked using the Levene test. Based on these results, either an independent-samples *t*-test or a U-Mann–Whitney test was employed for univariable analysis. Subsequently, subgroup analysis within the case group was conducted. The connection between the type of injury (intra-articular, extra-articular, or mixed) and the echogenicity and elasticity of PCFP, as well as the SCF/PCFP ratio, was assessed using the Kruskal–Wallis test. Post hoc analysis using the Dunn test was conducted based on the results. 

Parametric variables are reported as means with standard deviation (SD) and ranges, while non-parametric variables are presented as medians with interquartile ranges. Intraclass correlation coefficients (ICCs) for echogenicity measurements were calculated to assess intra- and inter-rater reliabilities. A significance level of *p* < 0.05 was considered statistically significant. All statistical calculations were performed using Statistica 13.3 software (StatSoft, Cracow, Poland), and detailed results are presented in tabular form.

## 3. Results

**Figure 4:**
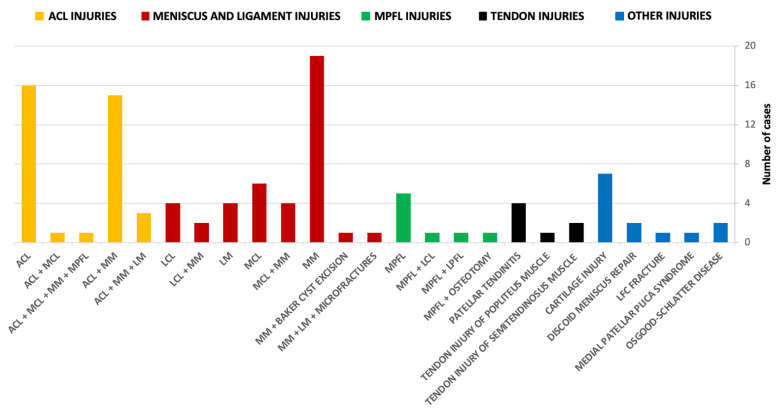
Epidemiology of knee pathologies or surgeries in the case group. ACL—anterior cruciate ligament, LCL—lateral collateral ligament, LFC—lateral femoral condyle, LM—lateral meniscus, LPFL—lateral patellofemoral ligament, MCL—medial collateral ligament, MM—medial meniscus, MPFL—medial patellofemoral ligament.

Initially, we enrolled a total of 110 knees across both groups. Three knees from the control group and four knees from the case group were excluded due to inclusion/exclusion criteria. Among these exclusions, two patients exhibited abnormalities in the ultrasonographic image of the PCL (indicative of prior PCL injury), three patients had a visible Baker’s cyst in MRI and/or ultrasound images, and two patients had grade III obesity, significantly complicating, and potentially distorting the ultrasound image due to the deep locations of the examined structures. The epidemiology of the knee pathologies or surgeries in the case group is presented in Figure 4. For the final analysis, we included 107 control knees and 106 case knees, resulting in dropout rates of 2.8% and 3.6%, respectively. 

Although women constituted 65% of the participants compared to 35% men, the groups were homogeneous in terms of sex distribution (*p* = 0.053). Among the participants, 4% were underweight, 67% had a normal BMI, 23% were overweight, and 6% had obesity class I. Both groups showed similarity in the mean BMI (*p* = 0.07). The sole statistically significant difference between the groups was in the mean age, with the case group being slightly older (*p* < 0.001). Detailed patient characteristics are presented in Table 2.

### 3.1. Sonographic Appearance of the PCFP

In all 107 assessed control knees in the US, the PCFP exhibited a homogenous appearance. It appeared triangular in shape and was situated between the PCL and the posterior knee capsule. Its echostructure appeared hyperechoic in comparison to the hypoechoic PCL, MHG, and SCF (Figure 2b). Unlike the SCF, the connective tissue septae were more challenging to differentiate from the fatty tissue lobules. The PCFP had its widest acoustic window in full extension and at a 30° knee flexion angle. However, due to the better visibility of the more tensed PCL at 30° flexion, this position was chosen for all measurements. As the flexion angle increased progressively (to 60° and 90°), the acoustic window decreased, making it more challenging to visualize the PCFP. At 120° of flexion, assessment was impossible for some more muscular patients. Longitudinal scans (in the oblique sagittal plane) were chosen over transverse scans, as they were deemed more representative and easier to interpret for measurements.

### 3.2. Echogenicity Measurements

The echogenicity of the PCFP inside the control group was significantly higher than PCL (*p* < 0.001, z-score = 8.97) and MHG (*p* = 0.007, z-score = 2.72) and lower than SCF (*p* < 0.001, z-score = −6.52). The echogenicity of the PCFP inside the case group was significantly higher than the rest of the structures (*p* < 0.001, z-score for PCL, MHG and SCF was 12.2; 11.65 and 12.36, respectively). The echogenicity of the PCFP in the case group was significantly higher than in the control group (*p* < 0.001, z-score = 8.78), and we achieved the same result with the ratio of SCF to PCFP between the groups (1.56 ± 0.61 controls vs. 0.44 ± 0.19 cases; *p* < 0.001; z-score = 12.37). The ICC for intra-rater agreement ranged from 76% to 94% for Rater 1 and from 83% to 97% for Rater 2, indicating good to excellent reliability. For inter-rater agreement, the ICC ranged from 72% to 97%, signifying values spanning from moderate to excellent reliability. Detailed ICC data are presented in Table 3 and Table 4.

### 3.3. Blood Flow Assessment

Blood flow was evaluated in all 107 knees from the control group. Blood flow was observed in 71% (76/107) of the PCFPs. Three variations of PCFP vascularity were identified: two-vesselled, one-vesselled, or lacking a visible vessel penetrating the fat pad from behind. The most prevalent vascularity pattern featured a single vessel, found in 50 out of the 107 controls. Among them, 6 controls showed one vessel in the superior part of the PCFP, while 44 exhibited it in the inferior part. Additionally, 26 controls displayed two visible vessels, while no vessels were identified in 31 controls.

### 3.4. Elasticity Measurements

In the control group, the elasticity of PCFP was lower than that of MHG and SCF, resulting in *p*-values of <0.001 (z-score = −12.64) and 0.005 (z-score = −2.83), respectively. Similarly, in the case group, the elasticity of PCFP was also lower than that of MHG and SCF, with both *p*-values being <0.001 (z-score for MHG was −12.58 and for SCF was −11.23). When comparing the elasticity of PCFP between the groups, we did not observe any significant differences in their values (*p* = 0.34, z-score = 0.95). However, the ratio of SCF/PCFP elasticity between the groups was higher in the case group (15.52 ± 17.87 vs. 2.26 ± 2.4; *p* < 0.001; z-score = 9.65), indicating a lower elasticity of PCFP among the knees in the case group.

### 3.5. Subgroups Analysis

The results of the Kruskal–Wallis test revealed a statistically significant difference among the subgroups concerning PCFP elasticity, with a calculated global *p*-value of 0.022. However, following a post hoc analysis using the Dunn test, none of the comparisons reached statistical significance. Additionally, the Kruskal–Wallis test indicated a statistically significant difference among the subgroups considering the elasticity ratio SCF/PCFP, with a calculated global *p*-value of 0.046. Nonetheless, after conducting a post hoc analysis using the Dunn test, none of the comparisons reached statistical significance. Detailed results can be found in Table 5 and Table 6. The subgroup analysis of the echogenicity of PCFP or the ratio SCF/PCFP did not show any significant differences, as evidenced by the global *p*-values of 0.216 and 0.772 in the Kruskal–Wallis test, respectively.

## 4. Discussion

The study delineates a structure that has been minimally covered in the literature: the pericruciate fat pad (PCFP). It presents a description of a normal, physiological PCFP, which holds clinical significance, as this understanding proves crucial during the most challenging aspect of knee ultrasound examinations—assessing the posterior compartment. Another crucial finding highlighted in the study is that, following trauma or surgery, the PCFP presents increased echogenicity and decreased elasticity. Detecting these variations in the PCFP during ultrasound examinations can assist in accurately evaluating this structure and prompt the examiner to exercise greater vigilance when changes in the PCFP are observed.

Quantitative ultrasound offers a secure, affordable, easily transportable, and comparatively available option for assessing tissue composition through imaging [40]. Post-acquisition image analysis using ImageJ software has been reported as a suitable tool for measuring tissue echogenicity [39]. It has been demonstrated to be valuable in assessing muscle quality and alterations in intramuscular adipose tissue, particularly within the skeletal muscle [40,41,44,45]. Strong correlations have been found between the echogenicity assessments in ultrasound and their CT [40] and MRI [41] equivalents. 

The echogenicity measurements of knee fat pads were utilized to distinguish between the two layers of Hoffa’s fat pad and the subcutaneous fat over the patellar tendon. There was a significant difference in echogenicity between the superficial and deeper HFP layers, with the deeper layer appearing brighter. However, no difference in echogenicity was observed between the superficial HFP layer and the subcutaneous fat [38]. Another notable study by Shibata et al. investigated the echogenicity of the PFP in healthy individuals and those with OA. The findings revealed significantly higher echogenicity in the OA group compared to the healthy group [21]. The authors suggest that increased echogenicity might be attributed to fibrosis within the PFP. This aligns with the outcomes of a study conducted by Pillen et al., wherein increased echogenicity in muscles was positively linked to the presence of fibrous tissue, as confirmed by histopathological examination [44].

To the authors’ knowledge, there have been no reports addressing the correlation between echogenicity alterations and knee injury/surgery specific to the PCFP. In our current study, we demonstrated that the echogenicity of the PCFP in the case group was notably higher than in the control group. Moreover, within the case group, the echogenicity of the PCFP was significantly higher compared to other structures, including the SCF. From the authors’ perspective, the rise in PCFP echogenicity could potentially be linked to fibrosis. However, the absence of histopathological examination remains a limitation and offers an intriguing path for future research. For example, in the future, taking a sample of the PCFP during ACL reconstruction or meniscal repair would be an interesting direction. For example, in the future, harvesting a sample of the PCFP during ACL reconstruction or meniscal repair would be an interesting direction to explore.

Besides bare echogenicity values, the authors also measured the SCF/PCFP echogenicity ratio. This method was previously introduced by Jeong et al. in their study on fatty infiltration of the supraspinatus muscle [32]. The authors adapted a method of assessment of the degree of fatty infiltration of the supraspinatus muscle on grayscale ultrasound images using a 3-point scale, with the echogenicity of the trapezius muscle serving as reference from the Khoury et al. [46]: grade 1, isoechogenic; grade 2, mildly hyperechogenic; grade 3, markedly hyperechogenic. Comparing the echogenicity of the target tissue with the surrounding tissues allows for extrapolation of the results of this study to other ultrasound machines and software. 

The preference for compression over shear-wave elastography was due to its greater accessibility, as well as to previous literature describing its usefulness in assessing elasticity of the adipose tissue [38]. To extrapolate the results of this study, the authors also measured the SCF/PCFP ratio. This method, previously introduced in the aforementioned study by Jeong et al. involved measuring the ratio of the elasticity of the trapezius to that of the supraspinatus muscle [32]. Comparing the elasticity of the target tissue with the surrounding tissues allows for a semi-quantitative assessment of elasticity when shear-wave elastography is unavailable [33]. 

The results of the current study underscore the importance of comparing ratios, as is commonly conducted in the literature [32,33,46], rather than focusing solely on raw values. This distinction is not immediately evident in the echogenicity results, since both the raw values of PCFP echogenicity and the SCF/PCFP ratio yielded statistically significant results. However, there were no significant differences observed in the elasticity of PCFP between the groups (*p* = 0.34). In contrast, the SCF/PCFP elasticity ratio was notably higher in the case group (15.52 ± 17.87 vs. 2.26 ± 2.4; *p* < 0.001; z-score 9.65), suggesting reduced PCFP elasticity among post-injured patients and underscoring the significance of employing ratios. Subgroup analyses revealed statistically significant differences in the PCFP elasticity (*p* = 0.022) and the SCF/PCFP elasticity ratio (*p* = 0.046). However, post hoc analyses, including both elasticity and echogenicity, indicated that none of the comparisons reached statistical significance, possibly due to underpowered groups in these analyses, which is further discussed as a limitation to these results. The deliberate omission of raw values in this study persists. Various ultrasound machines equipped with diverse parameters and software may yield disparate values. Consequently, comparing raw values remains of limited scientific merit.

Being aware of the functions of knee fat pads, it seemed reasonable to expect that a local intra-articular injury, such as an ACL injury [3] or chondral lesion [23] would prompt changes in the PCFP. However, the endocrine role of knee fat pads, led us to investigate whether the PCFP reacts in a systemic manner to injuries located outside the joint. Following subgroup analysis, overall values appeared promising. However, post-hoc analysis failed to reveal any statistically significant results. This phenomenon might stem from underpowered groups in these analyses, as the power analysis was computed only for the two primary groups (healthy and post-injured/surgical). Nonetheless, exploring populations with varying knee injury locations (intra-, extra-, or mixed) presents an intriguing avenue for future research. These findings contribute valuable data for future sample size analysis. The clinical significance of the PCFP’s response to injury can be leveraged in musculoskeletal diagnostics. For instance, assessing PCFP echogenicity, elasticity, or both, as well as developing a diagnostic tool akin to those created for ACL or cartilage injuries and predicting knee osteoarthritis using MRI [3,22,23], could represent an innovative approach to musculoskeletal diagnostics. This avenue holds promise, particularly considering the limitations of ultrasound in these conditions.

Thanks to Skaf et al., it has already been established that PCFP is rich in small blood vessels [1]. Subsequently, we confirmed that these vessels derive from the MGA, which enters the PCFP from behind [15]. Blood flow presence was detected in 71% (76/107) of the controls. The most frequent variation in PCFP vascularity appeared to be the presence of a visible vessel in the inferior part—41% (44/107). This finding is surprising because it contradicts previous literature. The entrance point of the MGA to the PCFP was described by Arthur et al. as the upper third of “a fatty tissue wrapped in a thin synovial membrane, which creases a triangular thickening” [13]. Nonetheless, this outcome underscores the necessity for additional caution during surgeries involving the posterior compartment within the knee joint. Due to the MGA and its branches, there is potential for PCFP to cause more swelling and bleeding than originally assumed. Awareness of variations in PCFP vascularity can be valuable during PCFP transfer techniques. Malinowski et al. suggested that preserving the lateral and distal peduncle of the fat pad is crucial when separating the PCFP from the anterior surface of the PCL. Our findings further contribute by revealing that, in rare instances (6 out of 107 PCFPs), it is the proximal peduncle that should be preserved.

The authors of this study acknowledge its limitations. Besides being a single-center retrospective study, the groups lacked complete homogeneity. Cases were slightly older, albeit still within the predefined inclusion criteria range, and there was an uneven distribution in terms of gender. The higher proportion of women over men stemmed from the fact that in the studied population—composed of medical and physiotherapy students—there were simply more women. This recruitment bias prevented statistical comparisons between genders. The convenience sampling method posed additional volunteer bias; however, this method was necessary due to organizational reasons. Following, knee MRIs were only ordered for 40% of the control group. Nonetheless, this proportion aligns with similar studies in the literature [38], and due to the comprehensive patient history and physical examinations conducted, this limitation was likely mitigated. The MRI assessments and ultrasound examinations were conducted by a single researcher, which may have introduced subjective bias. However, this approach also had a positive outcome, as it likely increased the homogeneity of these steps of the study. Nevertheless, it presents an interesting approach to conduct ultrasound examinations with two clinicians and independently evaluate the sonograms created by each of them. Lastly, the lack of histological confirmation of PCFP fibrosis following injury or surgical intervention remains the primary limitation of this study; however, like all the limitations, it sparks new ideas for future research. 

## 5. Conclusions

Our study presents novel findings indicating that, following knee trauma or surgery, the pericruciate fat pad (PCFP) experiences a significant increase in echogenicity and a concurrent decrease in elasticity. These changes suggest a potential association with fat pad fibrosis, which could hold diagnostic and monitoring value for knee pathologies. Moreover, our characterization of the vascularity variations supplying the PCFP highlights its crucial role as a conduit for the middle genicular artery, particularly relevant in surgical procedures like PCFP transfer techniques in ACL reconstruction or repair. By elucidating these attributes, our study underscores the clinical relevance of the PCFP in evaluating knee conditions, offering insights that could enhance our perspective on its diagnostic and clinical usefulness. Specifically, our findings suggest that changes in the echogenicity and elasticity of the PCFP may serve as valuable indicators for diagnosing and monitoring certain knee pathologies, such as ligament injuries or osteoarthritis. Understanding the diagnostic implications of these structural changes could lead to the development of more effective diagnostic protocols and treatment strategies, ultimately improving patient outcomes in orthopedic practice.

## Figures and Tables

**Table 1:** Parameters of sagittal and coronal T1W_TSE and sagittal and axial PDW_TSE_SPAIR_Tra sequences.

	Parameter	Frequency Bandwidth [kHz]	Echo Time [ms]	Repetition Time [ms]	Slice Thickness [mm]	Section Gap [mm]	Field of View [cm]	Acquisition Matrix	Number of Averages	Echo-Train Length
Sequence										
T1W_TSE		63.9	8	540.5	3.0	3.3	10 × 10	320 × 268	3	-
PDW_TSE_SPAIR_Tra		63.9	30	2591.5	3.0	3.3	10 × 10	320 × 256	1	12

**Table 2:** Detailed patient characteristics. BMI—body mass index, SD—standard deviation, M—males, F—females.

Variables	Tool	Controls	Cases	*p*-Value
BMI	Mean ± SD	22.91 ± 3.66	23.76 ± 3.10	=0.07
Age	Mean ± SD	21.94 ± 2.66	23.22 ± 2.66	<0.001
Sex	No.	M = 29; F = 78	M = 42; F = 64	=0.053

**Table 3:** Intra-rater assessment: PCFP—pericruciate fat pad, PCL—posterior cruciate ligament, MHG—medial head of gastrocnemius, SCF—subcutaneous fat.

	Structure	Rater 1	Rater 2
Controls	PCFP	0.93	0.97
	PCL	0.76	0.93
	MHG	0.77	0.97
	SCF	0.92	0.95
Cases	PCFP	0.92	0.93
	PCL	0.94	0.94
	MHG	0.93	0.76
	SCF	0.85	0.83

**Table 4:** Inter-rater assessment: PCFP—pericruciate fat pad, PCL—posterior cruciate ligament, MHG—medial head of gastrocnemius, SCF—subcutaneous fat.

	Rater 1 vs. Rater 2	Value
Controls	PCFP	0.97
	PCL	0.94
	MHG	0.92
	SCF	0.95
Cases	PCFP	0.93
	PCL	0.97
	MHG	0.92
	SCF	0.72

**Table 5:** Results of subgroup analysis of the case group assessing the elasticity of the PCFP.

	Extra-Articular	Intra-Articular	Mixed
Extra-articular	-	0.070	0.447
Intra-articular	0.070	-	<1.000
Mixed	0.447	<1.000	-

**Table 6:** Results of subgroup analysis of the case group assessing the elasticity ratio SCF/PCFP.

	Extra-Articular	Intra-Articular	Mixed
Extra-articular	-	0.110	0.105
Intra-articular	0.110	-	<1.000
Mixed	0.105	<1.000	-

## Data Availability

Data are contained within the article.
